# The Tumor Suppressor Protein TRAF3 Modulates GSK3 Activity and Susceptibility of B Lymphoma Cells to GSK3 Inhibition

**DOI:** 10.3390/cancers14205029

**Published:** 2022-10-14

**Authors:** Emma L. Hornick, Laura L. Stunz, Shakoora Sabree, Xiaosheng Wu, Thomas E. Witzig, Gail A. Bishop

**Affiliations:** 1 Department of Microbiology and Immunology, The University of Iowa, Iowa City, IA 52242, USA; 2 Veterans Administration Medical Center, Iowa City, IA 52242, USA; 3 Graduate Program in Immunology and MSTP Program, The University of Iowa, Iowa City, IA 52242, USA; 4 Division of Hematology, Department of Medicine, Mayo Clinic, Rochester, MN 55905, USA

**Keywords:** B cell lymphoma, B cell survival, lymphomagenesis, GSK3, TRAF3

## Abstract

**Simple Summary:**

TRAF3 is an adapter protein that regulates signals through many receptors important for B cell differentiation and function. Loss-of-function mutations or deletions of TRAF3 are common in B cell malignancies. Glycogen Synthase Kinase 3 (GSK3) regulates signaling in growth, survival, and metabolism pathways. GSK3 inhibitors have been effective against many solid tumors; the inhibitor used in this work is currently being tested for efficacy against BCLs. We found that TRAF3 and GSK3 associate in multiple BCL cell lines, and that BCLs with low TRAF3 have a higher susceptibility to GSK3 inhibition. In contrast to BCL cell lines, GSK3 inhibition has little effect on TRAF3-sufficient and deficient resting primary B cells. These results suggest TRAF3 level as a predictor of BCL responsiveness to GSK3 inhibitor therapy.

**Abstract:**

TNF receptor-associated factor 3 (TRAF3) is an adapter protein that inhibits many signals that promote B cell survival and activation. Mice with a B cell-specific TRAF3 deficiency and humans with a rare haploinsufficiency in TRAF3 have enhanced development of BCLs as they age. Loss-of-function mutations in TRAF3 are common in B cell malignancies. Recent studies show that pharmacological inhibition of the enzyme glycogen synthase kinase 3 (GSK3), which regulates cellular growth, survival, and metabolism, inhibits growth and survival of BCL-derived B cells. In this study, we found that TRAF3 and GSK3 associated in B cells. The relative levels of TRAF3 in BCL cell lines correlated positively with the ratio of inactive to total GSK3β, and negatively correlated with susceptibility to GSK3 inhibition by the GSK3 inhibitory drug 9-ING-41, currently in clinical trials. Uniquely in BCLs with low TRAF3, GSK3 inhibition caused increased loss of the TRAF3-regulated, anti-apoptotic protein Mcl-1. GSK3 inhibition also blocked hyperresponsiveness to IL-6 receptor signaling in TRAF3-deficient BCL cells. Together, these results support the utility of 9-ING-41 as a treatment for BCL, and suggest that a decrease or loss of TRAF3 in BCLs could act as a biomarker for increased susceptibility to GSK3 inhibitor treatment.

## 1. Introduction

B cell lymphomas (BCLs) often achieve unchecked survival and proliferation via chronic signaling through pathways that drive activation and differentiation in normal B cells [1]. Many of these pathways converge upon NF-κB activation, and removal or blockade of negative regulators of NF-κB is one of the most common means by which BCLs evade programmed cell death [1]. The TNF receptor-associated factor (TRAF) family of proteins regulate NF-κB activation at different levels downstream of antigen, cytokine, and pattern recognition receptors [2]. TRAF3 is a TRAF family member with diverse cell- and receptor-type specific functions that include negative regulation of signaling cascades promoting B cell activation, differentiation, and survival [3]. TRAF3-deficient B cells exhibit constitutive NF-κB2 activation [4], amplified IL-6R signaling leading to increased plasma cell differentiation [5], enhanced glucose uptake and metabolism [6], and increased B cell receptor (BCR) and Toll-like receptor (TLR) signaling [7,8]. Additionally, TRAF3-deficient B cells display strikingly increased homeostatic survival compared with wild-type (WT) B cells [4]. The cumulative effect of B cell-restricted TRAF3 deficiency in mice (B-*Traf^−/−^*) is early manifestations of autoimmunity and later development of BCLs [4,9]. This phenotype is highly clinically relevant, as malignancy-associated functional and genetic losses of human TRAF3 at both the genetic and post-translational levels have also been reported. Several independent groups identified homozygous deletion or inactivating mutations of *TRAF3* in human B cell-derived malignancies [10,11,12,13], and our prior work showed that B cells can be rendered functionally TRAF3-deficient by TRAF3 sequestration via association with the Epstein–Barr Virus-encoded oncoprotein Latent Membrane Protein 1 (LMP1). LMP1 is a CD40 mimic that binds TRAF3 with much higher avidity than CD40, effectively depleting the cellular pool of TRAF3 [14,15]. This TRAF3 sequestration by LMP1 interferes with TRAF3-mediated negative regulation of homeostatic survival and may be a key mechanism involved in malignant transformation of EBV^+^ BCLs [15]. One of the biggest challenges in treating BCL is drug resistance leading to relapse, which may be pre-empted with better biomarkers that predict BCL susceptibility to initial treatments. A thorough understanding of TRAF3-mediated regulation of pathways that BCL cells exploit for survival and pathogenesis will enable better predictions of response based on TRAF3 expression status of a particular BCL.

Glycogen Synthase Kinase 3 (GSK3) is a ubiquitously expressed serine/threonine kinase with both alpha and beta isoforms. GSK3 kinases have at least 40 substrates involved in essential cellular processes including growth, proliferation, apoptosis and metabolism [16,17]. GSK3 inhibition has been effective at halting solid tumor growth and has recently emerged as a promising treatment for BCL [18,19,20]. In normal mature B cells, GSK3 promotes quiescence and homeostasis while restraining cell mass accumulation and proliferation in germinal center B cells, allowing these cells to tolerate nutrient-limiting conditions of the dark zone [21,22]. Phosphorylation-mediated inhibition of nuclear GSK3 protects activated B cells from cell death pathways triggered by double-stranded DNA breaks as they undergo Ig class-switch recombination [23]. Despite what seem to be tumor-suppressive roles for GSK3 in primary B cells, pharmacological GSK3 inhibition effectively halts growth of many BCL cell lines, suggesting that GSK3 may play a distinct role in B cells that have undergone malignant transformation. This phenomenon of ‘oncogene-addiction’ is well-documented and is seen in multiple TRAF3-regulated B cell-specific pathways as well [6,24,25].

GSK3 participates in several of the pathways modulated by TRAF3 in B cells, thus we investigated the interplay between TRAF3 and GSK3 in BCL-derived human cell lines and primary B cells. GSK3β, but not GSK3α, has a predicted TRAF3 binding site and indeed, we found that GSK3β associated with TRAF3 in multiple BCL lines. The relative amount of TRAF3 in BCL lines was positively correlated with the ratio of inactive to total GSK3β, and negatively correlated with susceptibility to GSK3 inhibition by CHIR-99021 and the drug 9-ING-41, currently in clinical cancer trials (NCT03678883). Susceptibility of TRAF3 low or negative BCL to 9-ING-41 was accompanied by increased loss of the anti-apoptotic molecule Mcl1, which is elevated in TRAF3-deficient primary B cells and contributes to their enhanced homeostatic survival [24]. Interestingly, GSK3 inhibition also reversed hyper-responsiveness to IL-6 receptor signaling in a TRAF3-deficient BCL line compared to the TRAF3-sufficient parent line. Consistent with previous work [19], we detected little or no effect of GSK3 inhibition by 9-ING-41 on primary B cells, regardless of TRAF3 status. Our findings support the utility of 9-ING-41 as a treatment for BCL and suggest that a decrease or loss of TRAF3 in BCLs could act as a biomarker for increased susceptibility to GSK3 inhibitor treatment.

## 2. Materials and Methods

### 2.1. Mice

Generation of mice with a B cell-specific TRAF3 deficiency (*Cd19*-Cre^+/−^*Traf3^flox/^*^flox^) was performed as previously described [4]. B cells from littermate controls, *Cd19*-Cre^+/−^*Traf3^+/+^* or *Cd19*-Cre^−/−^*Traf3^flox/flox^* are referred to throughout as “WT”. Mice were maintained under specific pathogen free conditions and used in accordance with the National Institute of Health guidelines under animal protocols 1990801 and 1062397, approved by the Animal Care and Use Committee at the University of Iowa. Both male and female mice aged 2–6 mos were used in similar numbers in these studies. 

### 2.2. Cells and Treatment

Splenic mouse B cells were enriched using a negative selection kit (Stemcell Technologies, Vancouver, BC, Canada). Peripheral blood mononuclear cells were obtained from human whole blood leukocyte reduction cones obtained from DeGowin Blood Center at the University of Iowa. Healthy donors 17 to 70 years of age provided written consent for their blood to be used in research projects in compliance with the University of Iowa’s Institutional Review Board. Mononuclear cells were isolated by centrifugation in Histopaque 1077 (Invitrogen, Waltham, MA, USA), and B cells were enriched with a negative selection kit (Stemcell Technologies). Primary mouse B cells, mouse B cell lymphoma cell lines, and human B cell lymphoma cell lines were cultured in complete medium: RPMI 1640 (Life Technologies) containing 10% heat-inactivated fetal calf serum, 10 μM β-mercaptoethanol (Sigma, St. Louis, MO, USA), 2 mM L-Glutamine (Gibco, Waltham, MA, USA) and 100 U/mL penicillin-streptomycin (Gibco). Provenance of cell lines is as follows: A20.2J [26] and *Traf3^−/−^* A20.2J [14] were generated in our laboratory; BJAB [27] was purchased from ThermoFisher Scientific, Dawo [28] and Karpas422 [29] were provided by the George Weiner laboratory at the University of Iowa; OCILy1, OCILy3, DHL-6, GSK3-deficient OCILy1 subclones AB199-1 and AB12-12, and OCILy7 [30] was provided by the Thomas Witzig laboratory at the Mayo Clinic in Rocherster, MN; Daudi [31], Ramos [32], Ramos 2G6 [32], and SKW 6.4 [33] were purchased from ATCC (Manassas, VA, USA); and T5-1 [34] was purchased from Sigma. TRAF3-deficient BJAB subclones were generated as previously described for the HuT28.11 cell line [35]. Briefly, guide RNA/Cas9 vector constructs (pX330) for disruption of two segments of the *TRAF3* gene were prepared using the CRISPR design tool (crispr.mit.edu) maintained by Dr. Feng Zhang. The double-stranded synthetic oligonucleotides 5′ CACCGCCATCATATCCTCTCATGCA 3′, and 5′ AAACTGCATGAGAGGATATGATGGC 3′ were used to target intron one. Exon five was targeted with the following pairs: 5′ CACCGGTTCCGATGATCGCGCTGC 3′ and 5′ AAACGCAGCGCGATCATCGGAACC 3′. BJAB cells suspended in Optimem (Invitrogen) were transfected by electroporation at 225 V for 30 ms in the presence of: 2.5 μg of each of the two guide RNA/Cas9 vectors, 0.5 μg pEGFP-C1 (Clontech, Mountain View, CA, USA), and 5 μg double-stranded filler DNA oligonucleotides (random sequence). Twenty-four hours later, GFP-expressing cells were sorted using a Becton Dickinson FACS Fusion and plated at 1 cell/well into 96-well plates. Clones were screened by PCR of genomic DNA using the following primers: *TRAF3* targeting primers include 5′ CTGAAAGACAGCAGGTCTCAGGCAC 3′, and 5′ GAATGTATCATATAGGAATTGAGTGG 3′ (Int-5R3), and by Western blotting. GSK3 inhibitors CHIR-99021 (Sigma) and 9-ING-41 (generously provided to T. E. W. by Daniel Schmitt, Actuate Therapeutics, Inc.) were reconstituted in DMSO. All experiments including inhibitor treatments also included control groups with equivalent concentrations (*v*/*v*) of DMSO only. Survival of primary B cells and BCL cell lines during GSK3 inhibitor treatment was quantified by propidium iodide exclusion. Where indicated, 20 μg/mL LPS (Invivogen, San Diego, CA, USA) was added to primary mouse B cell cultures 2 h prior to addition of 9-ING-41 for survival analysis. For IL-6 receptor (IL-6R) signaling studies, cells were pre-treated with 5 μM 9-ING-41 for two hours followed by stimulation with 20 ng/mL IL-6 (Peprotech, Cranbury, NJ, USA) in the presence of 5 μM 9-ING-41. 

### 2.3. Immunoprecipitation

Cells were lysed in Immunoprecipitation (IP) lysis buffer (40 mM Tris pH 7.5, 0.5% Triton-X-100, 100 mM NaCl, 1 mM MgCl_2_, 1 mM CaCl_2_, 2 mM Na_3_VO_4_, and EDTA-free cOmplete mini protease inhibitor cocktail (Roche, Basel, Switzerland)) on ice for 30 min. Lysates were pre-cleared for 10 min with protein G Dynabeads (Invitrogen). Antibody (Ab) recognizing one of the following was added: TRAF3 (Santa Cruz Biotechnology (Dallas, TX, USA) #sc-1828, 1:1000, or Cell Signaling Technologies (CST, Danvers, MA, USA) Ab #4729, 1:100) or GSK3β (CST, #12456). Lysates were incubated with IP Ab for 2 h at 4 °C, then Ab-bound targets were captured with protein G Dynabeads for 20 min at 4 °C. Dynabeads were washed 3X with IP washing buffer (20 mM Tris pH 7.5, 1% Triton-X-100, 40 mM NaCl) then resuspended in IP lysis buffer for Western blotting analysis.

### 2.4. Western Blotting

Cell lysates were prepared in 1X Radio Immunoprecipitation Assay (RIPA, CST #9806) Buffer containing EDTA-free cOmplete mini protease inhibitor cocktail (Roche). Proteins were separated on a NuPAGE gel (Invitrogen) and transferred to a polyvinylidene difluoride membrane using the XCell II blotting system (Invitrogen), following manufacturer recommendations. Membranes were blocked with 5% nonfat milk and incubated with 1° Ab overnight at 4 °C. The following 1° Abs were used for Western blotting: Cell Signaling Technologies: GSK3β (#12456), phospho-GSK3β^S9^ (#5558), Mcl1 (#94296), phospho-STAT3^Y705^ (#9145) STAT3 (#4904), TRAF3 (#4729); Santa Cruz Biotechnology: β-Actin (#sc-47778), GAPDH (#sc-47724). Following 1° Ab incubation, membranes were washed and incubated with HRP-tagged goat anti-mouse IgG (Jackson Immunoresearch Labs, Westgrove, PA, USA, #115-035-003) or HRP-tagged goat anti-rabbit IgG (Jackson Immunoresearch Labs, #111-035-144) for 2 h at room temperature. Blots were imaged using SuperSignal West Pico or Femto substrate (Thermo Fisher Scientific, Waltham, MA) and a low-light imaging system (LAS400, Fuji Medical Imaging, Tokyo, Japan). Densitometry of Western blots was performed in ImageStudio Lite software (LI-COR Biosciences, Lincoln, NE, USA) or MultiGauge Imaging software (Fuji Medical Imaging). Density of bands for proteins of interest was divided by density for loading control (Actin or GAPDH) from the same sample to normalize protein levels.

### 2.5. Statistical Analysis

Data were graphed and the indicated statistical tests performed using GraphPad Prism software.

## 3. Results

### 3.1. TRAF3 Association with GSK3β in BCL-Derived B Cells

**Figure 1:**
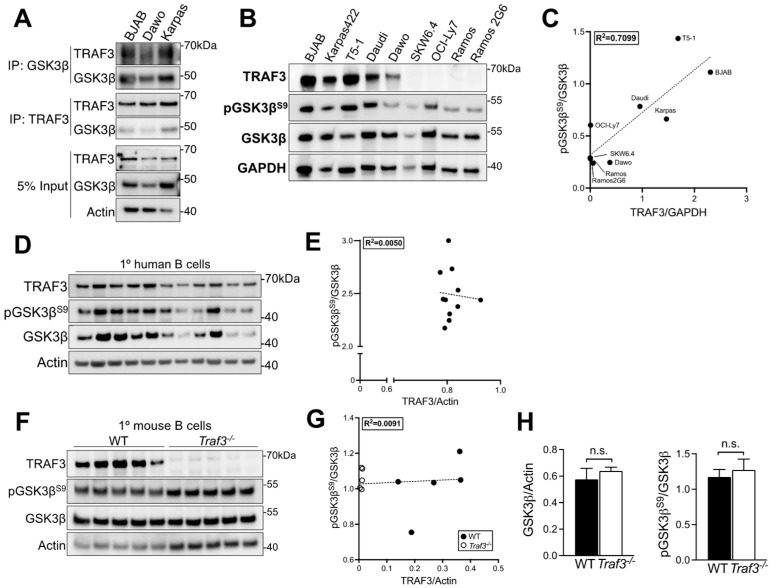
Relationship between TRAF3 status and inactive GSK3β in B cell lymphoma cell lines. (**A**) Cells from BCL cell lines were lysed and GSK3β or TRAF3 was immunoprecipitated. IP samples and input/whole cell lysate (WCL) were analyzed by Western blotting. (**B**) Representative blot of TRAF3, GSK3β and pGSK3β^S9^ in a panel of BCL cell lines. (**C**) Graph of amount of TRAF3 vs. ratio of inactive (pGSK3β):total GSK3β from data in (**B**). (**D**) Western blot of human B cells from the peripheral blood of healthy donors. (**E**) Graph of amount of TRAF3 vs. ratio of inactive (pGSK3β):total GSK3β from data in (**F**). Representative Western blot of pGSK3β^S9^ and total GSK3β in primary mouse B cells from mice with or without a B cell-specific TRAF3 deficiency. (**G**) Graph of amount of TRAF3 vs. ratio of inactive (pGSK3β):total GSK3β from data in (**F**). (**H**) Quantification of blots in (**D**). Blots are representative of at least 3 independent experiments (**A**,**B**). In (**C**), each lane represents an individual human donor. In (**D**), each lane represents an individual mouse. n.s., not significant by student’s *t*-test. Uncropped blots and densitometry shown in Figures S3 and S4.

TRAF3 regulates many aspects of B cell biology. These include several pathways restraining homeostatic survival, as well as signaling by the B cell antigen receptor (BCR), various tumor necrosis factor receptor superfamily members, Toll-like receptors (TLRs) and cytokine receptors [3]. Dysregulation of these pathways can make important contributions to malignant transformation of B cells, and indeed, mice with a B cell-specific *Traf3* deletion develop spontaneous BCLs [9]. The pleiotropic enzyme glycogen synthase kinase 3 (GSK3) is also an important regulator of activated B cells and was recently identified as a potentially promising therapeutic target for BCLs [19]. We therefore investigated the possibility of a BCL-relevant relationship between TRAF3 and GSK3 in malignant B cells. TRAF3 has several GSK3 substrate consensus sequences, but only the β isoform of GSK3 has predicted TRAF3 interaction sites. Consistent with a previous study [36], we found that TRAF3 co-immunoprecipitated with GSK3β in three BCL cell lines: BJAB, a Burkitt Lymphoma; Karpas 422, a Germinal Center B cell-like Diffuse Large B Cell Lymphoma (GCB DLBCL), and Dawo, a DLBCL not otherwise specified (Figure 1A). This stimulated further investigation of how TRAF3 impacts GSK3β function.

### 3.2. TRAF3 Regulation of the Ratio of Inactive: Total GSK3β in Malignant B Cells

Spontaneous genetic loss of TRAF3 in BCLs has been reported in both humans and dogs [10], but variation in the amount of TRAF3 protein in malignant B cells may also arise post-translationally and cause clinically significant phenotypic changes [15]. We compared the levels of TRAF3, inactive GSK3β (i.e., GSK3β phosphorylated at Ser9), and total GSK3β in multiple BCL cell lines, and found a linear correlation between relative amount of TRAF3 protein and the ratio of inactive:total GSK3β (Figure 1B,C and Figure S1). Interestingly, neither primary B cells from healthy human blood donors or young mice with a B cell-specific TRAF3 deficiency showed a correlation between the ratio of inactive:active GSK3β and the relative amount of TRAF3 (Figure 1D–H). This suggests that TRAF3 deficiency impacts GSK3β activity during or after malignant transformation of B cells.

### 3.3. Impact of TRAF3 on Susceptibility of B Cells to GSK3-Mediated Inhibition of Survival

**Figure 2:**
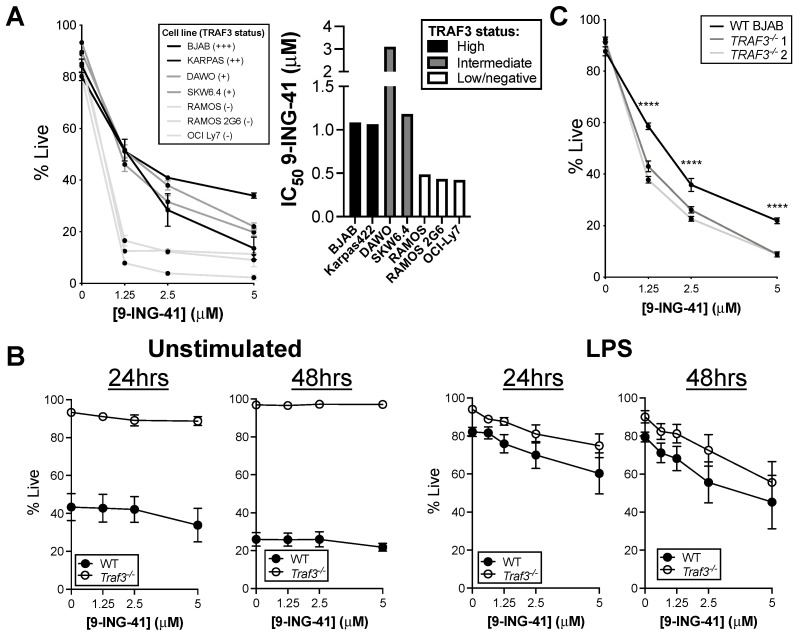
TRAF3 status of BCL cell lines affects susceptibility to GSK3 inhibition. (**A**) Survival of BCL cell lines after 48 h incubation with indicated concentrations of 9-ING-41 (left) and IC_50_ calculated from survival data (right). (**B**) Survival of WT or *Traf3^−/−^* primary mouse B cells after 24 or 48 h incubation with indicated concentrations of 9-ING-41. In right two graphs, cells were activated with LPS 2 h prior to initiation of 9-ING-41 treatment. (**C**) Survival of BCL cell line BJAB and two clones of TRAF3-deficient BJAB after 48 h incubation with indicated concentrations of 9-ING-41. Graphed are mean ± SEM for at least three independent replicates. **** *p* < 0.0001 by repeated measures ANOVA with Sidak’s multiple comparisons test.

Recent work showed efficacy of the GSK3 inhibitor 9-ING-41 in halting BCL cell cycle progression in vitro and in limiting growth of a lymphoma xenograft in vivo [19]. Given the correlation between inactive:total GSK3β and TRAF3 in BCL lines, we tested whether relative TRAF3 protein levels would predict susceptibility to GSK3 inhibition by 9-ING-41. Consistent with our earlier studies, this inhibitor was very effective at killing BCL cell lines within 48 h (Figure 2A). Strikingly, BCL-derived cells with low or undetectable TRAF3 were more susceptible than those with high TRAF3 to 9-ING-41-mediated killing (Figure 2A). We confirmed a similar pattern of susceptibility by TRAF3 level to the GSK3 inhibitor CHIR-99021 (Figure S2). We also tested the effect of GSK3 inhibition on TRAF3-deficient primary mouse B cell and littermate control B cell survival and found no differential effect or dose-responsiveness (Figure 2B). The resistance of resting primary mouse B cells to GSK3 inhibition is consistent with the similar finding that resting human B cells from healthy donors are impervious to 9-ING-41 treatment in vitro [19]. GSK3β in B cells is particularly important during periods of high metabolic activity and active growth, e.g., germinal center reactions [21,22], which may explain why resting B cells are unaffected by its inhibition. We thus tested whether inhibition of GSK3 would affect survival of B cells responding to LPS stimulation. B cells treated with LPS showed dose-responsive sensitivity to 9-ING-41 after 24 and 48 h after LPS treatment, indicating that activated B cells are more vulnerable to GSK3 inhibition than resting B cells (Figure 2B). There was no significant difference in killing of primary mouse B cells based on TRAF3 status at 24 h after LPS stimulation, but littermate controls showed somewhat higher susceptibility to killing by 48 h of treatment. Together, these data indicate that transformed B cell lines and activated B cells were vulnerable to GSK3 inhibition, particularly transformed cells with low or no TRAF3 protein. However, TRAF3 status had a very minor effect on susceptibility of primary B cells to GSK3 inhibition, even upon activation.

Primary BCLs and BCL-derived cell lines exhibit a high degree of heterogeneity, so it was possible that factors besides TRAF3 status were affecting the responsiveness of these cell lines to 9-ING-41 treatment. We thus generated a TRAF3-deficient version of the human BCL line BJAB, which normally has a high level of TRAF3, to more specifically test the effect of TRAF3 level on susceptibility to GSK3 inhibition. Two distinct clones of TRAF3-deficient BJAB cells showed significantly lower tolerance for GSK3 inhibition by 9-ING-41, arguing that loss of TRAF3 alone is sufficient to increase BCL susceptibility to killing by GSK3 inhibition (Figure 2C).

### 3.4. Involvement of Mcl1 in How TRAF3 Status Predicts 9-ING-41 Susceptibility of BCL Cells

**Figure 3:**
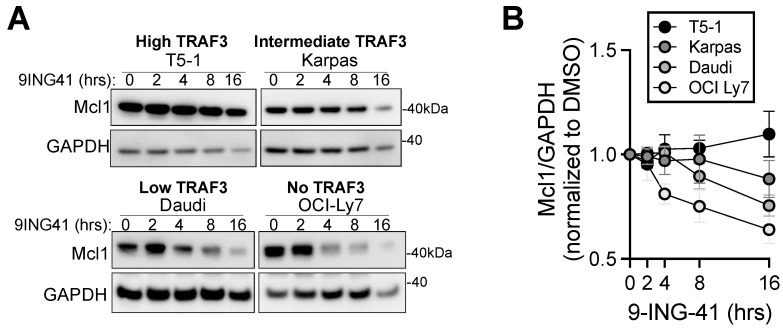
Effect of GSK3 inhibition on Mcl1 degradation in BCL cell lines. (**A**) Representative Western blots of Mcl1 over time of 9-ING-41 treatment in the indicated BCL cell lines. (**B**) Mcl1 and GAPDH were quantified by densitometry and the ratio relative to DMSO treated cells is graphed. Blots in (**A**) are representative of 3 independent experiments, whose results are all graphed as mean ± SEM in (**B**). Uncropped blots and densitometry shown in Figure S5.

One of the mechanisms by which TRAF3 restrains homeostatic B cell survival is by targeting the transcription factor cycling AMP-responsive element binding protein (CREB) for degradation in the nucleus [24]. In B cells lacking TRAF3, CREB target genes are upregulated, including the gene encoding pro-survival Bcl2 family member Mcl1. Data discussed above indicated that TRAF3-deficient BCL-derived cell lines were especially susceptible to 9-ING-41-mediated killing, thus we investigated whether this was related to Mcl1 levels, which are affected by TRAF3 status in primary B cells [24]. Indeed, we saw a decrease in Mcl1 protein upon 9-ING-41 treatment that was inversely related to the amount of TRAF3 expressed in the BCL cell line (Figure 3A,B). That is, BCL lines with low or no TRAF3 had 30–40% less Mcl1 relative to total protein after 16 h of treatment with 9-ING-41, compared to BCL lines with high or intermediate TRAF3, which lost no more than 15% of their Mcl1 relative to total protein. These data suggest that enhanced susceptibility to GSK3 inhibition in BCL cell lines with low or no TRAF3 involves increased reliance on Mcl1-dependent survival pathways. This conclusion is supported by our previous findings, showing that TRAF3-deficient B cells are particularly susceptible to killing via CREB inhibition [24].

### 3.5. Correction of Enhanced IL-6 Receptor Signaling in TRAF3-Deficient Malignant B Cells via GSK3 Inhibition

**Figure 4:**
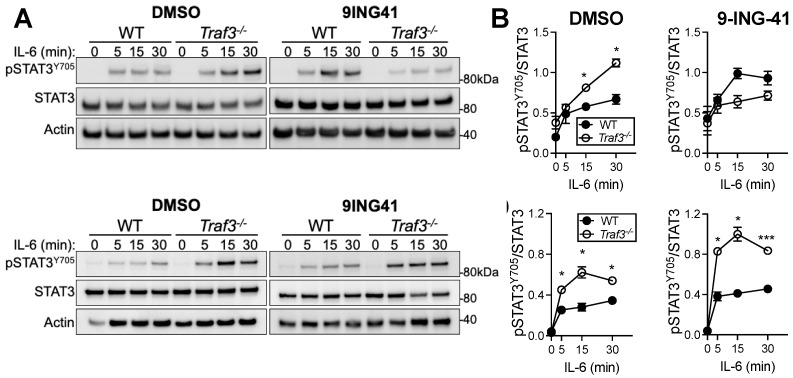
Differential effect of GSK3 inhibition on IL-6-induced STAT3 activation. (**A**) Representative Western blot of IL-6-induced pSTAT3^Y705^ in 9-ING-41 or DMSO-treated mouse BCL cells (A20.2J and *Traf3^−/−^* A20.2J). (**B**) Quantification of 4 replicates including A. (**C**) Representative Western blot of IL-6-induced pSTAT3^Y705^ in 9-ING-41 or DMSO-treated primary WT or *Traf3^−/−^* mouse B cells. (**D**) Quantification of 3 replicates including C. Data points are mean ±SEM. *** *p* < 0.001, * *p* < 0.05 by repeated-measures ANOVA with Sidak’s multiple comparisons test. Uncropped blots and densitometry shown in Figures S6 and S7.

We previously reported that mice with a B cell-specific TRAF3 deficiency have a significantly expanded plasma cell compartment, which is dependent on enhanced signaling by IL-6R [5]. Interestingly, many of these mice develop BCL in their first year of life [4]. TRAF3 dampens IL-6R signaling through its recruitment of the negative regulatory phosphatase PTPN22 to IL-6R-associated Janus Kinase (JAK) family member JAK1 [5]. TRAF3-deficient B cells thus show exaggerated activation of JAK1 and its transcription factor target STAT3 in response to IL-6 stimulation, leading to the enhanced accumulation of plasma cells, which is also observed in PTPN22-deficient mice [5]. Increased activated STAT3 is also responsible for the upregulation of pro-survival kinase Pim2 in TRAF3-deficient B cells, though this may be independent of IL-6R signaling [25]. Together, these findings emphasize the importance of IL-6 and STAT3 in multiple tumor-suppressive functions of TRAF3. Therefore, we tested the effect of GSK3 inhibition on the increased STAT3 activation phenotype of a TRAF3-deficient mouse BCL-derived cell line, A20, and TRAF3-deficient primary B cells. In the BCL cells, DMSO (diluent control)-treated TRAF3-deficient cells showed the characteristic pattern of enhanced IL-6-induced pSTAT3^Y705^ compared to TRAF3-sufficient cells (Figure 4A,B). In contrast, the cells treated with GSK3 inhibitor 9-ING-41 showed a complete reversal of the enhanced pSTAT3^Y705^ phenotype upon IL-6 stimulation. In primary B cells lacking TRAF3, 9-ING-41 treatment failed to ameliorate the increase in IL-6-induced pSTAT3^Y705^, consistent with the trend of primary B cell resistance to the effects of GSK3 inhibition (Figure 4C,D).

Together, the data presented here support a model in which loss of TRAF3 renders B lymphoma cells more susceptible to killing by GSK3 inhibition, whereas primary B cells are unaffected. The increased death experienced by 9-ING-41-treated TRAF3-deficient BCL cells is likely at least in part caused by an increased loss of Mcl1, and/or to the loss of their hyper-responsiveness to IL-6 signaling. These findings build upon our previous report showing that 9-ING-41 is not toxic to resting normal B cells, and importantly, indicate that TRAF3-deficient BCLs are more likely to respond well to GSK3 inhibition.

## 4. Discussion

TRAF3 is an important regulator of many central pathways in B cell biology. These include different pathways that promote homeostatic survival and glucose metabolism, as well as multiple important receptor complexes present on B cells: the BCR, members of the TNFR superfamily (e.g., CD40, BAFF receptor), TLRs and the IL-6R [3]. Loss of TRAF3 renders unstimulated B cells hypermetabolic and confers a significant homeostatic survival advantage, characteristics reminiscent of malignant B cells. Additionally, BCR, TLR and IL-6R-mediated effects are implicated in the pathogenesis of B cell malignancies [1]. In support of this important impact of TRAF3 deficiency upon the pre-malignant cell phenotype, both functional and genetic loss of TRAF3 have been reported in B cell malignancies, and mice with a B cell specific TRAF3 deficiency have an increased incidence of lymphomas later in life [9,37,38].

Though long appreciated for its roles in cell growth and metabolism, the multi-functional kinase GSK3 has only recently been appreciated for its importance as a metabolic regulator in B cells [21], and still more recently as a promising therapeutic target for BCL [19,20]. Shortly after the initial study describing the B cell-specific role of GSK3, two groups including our own described GSK3 inhibition by the drug 9-ING-41 as effective at halting the growth of BCL cells in vitro and in vivo [19,39]. Interestingly, there was a range of susceptibility to GSK3 inhibition among different BCL-derived cell lines and patient samples. We therefore investigated TRAF3 status as a possible important modifier of BCL susceptibility to GSK3 inhibition.

In this study, we report an interesting negative correlation between the ratio of inactive to total GSK3β and the amount of TRAF3 cellular protein across several different types of BCL cell lines, but not in primary mouse B cells. Consistent with the reduced amount of inactive GSK3 in BCL cell lines with low or no TRAF3, these cell lines were more susceptible to killing with both the promising new GSK3 inhibitor 9-ING-41, and CHIR-99021, an older GSK3 inhibitor. Previous studies showed no effect of GSK3 inhibition on primary B cell survival, and indeed we saw little effect of 9-ING-41 treatment on survival of resting B cells. There was also only a minimal differential effect of TRAF3 status on survival following 9-ING-41 treatment in primary mouse B cells. We confirmed that the differential susceptibility of BCL cell lines was likely attributable to TRAF3 by using CRISPR/Cas9 to delete TRAF3 from the BCL cell line BJAB. TRAF3 deficiency significantly decreased the IC_50_ of 9-ING-41 of two different subclones of TRAF3-deficient BJAB cells compared to the parent line. Among BCL cell lines with naturally varied levels of TRAF3, we observed a greater decline in the anti-apoptotic molecule Mcl1 in TRAF3 low or negative BCL cell lines compared to those with an intermediate or high level of TRAF3. Finally, we explored the possibility that GSK3 inhibition could differentially affect other TRAF3-regulated pathways based on TRAF3 status. TRAF3 regulates IL-6R signaling through its recruitment of the inhibitory phosphatase PTPN22, which dampens IL-6-induced JAK1 and STAT3 activation [5]. In TRAF3-deficient primary mouse B cells and mouse BCL cell lines, PTPN22 is unable to reach the IL-6R complex and exert its negative regulatory effect, resulting in exaggerated IL-6R signaling. We found that 9-ING-41 treatment of a WT and TRAF3-deficient mouse BCL cell line corrected the exaggerated IL-6-induced STAT3 phosphorylation in the TRAF3-deficient cells. However, the enhanced IL-6R signaling in TRAF3-deficient primary mouse B cells was not affected by 9-ING-41 treatment. This finding suggested that TRAF3 status only affects responsiveness to GSK3 inhibition in B cells that have undergone malignant transformation.

A high priority for future studies is assessment of how TRAF3 status can predict other therapies likely to synergize with GSK3 inhibition. The study that initially described the efficacy with which GSK3 inhibition kills BCL cells tested GSK3 inhibition in combination with other inhibitors but found no universally successful combination [39]. This is understandable due to the diverse mechanisms of survival employed by different BCL subtypes and individual clones. Data presented here support using TRAF3 status to help address this clinical challenge, and better predict effective synergistic combinations for a given BCL. In addition, staining for TRAF3 on BCL biopsies from patients entering trials of 9-ING-41 should be performed to learn if this indeed is a clinical predictive biomarker. As discussed above, TRAF3 regulates many pathways co-opted by BCL cells for their enhanced survival [1]. We can synthesize our understanding of TRAF3-deficient B cell phenotypes and pathways central to survival for a given BCL subtype to identify more effective initial therapies. For example, our group recently reported a positive correlation between TRAF3 levels and susceptibility to the BTK inhibitor ibrutinib in several BCL cell lines [40]. It is therefore possible that combining these two inhibitors would have a synergistic effect in TRAF3 low or negative BCLs. TRAF3-deficient B cells also have elevated levels of the pro-survival kinase Pim2, which contributes to their enhanced homeostatic survival [25]. Pim2 inhibitors were promising chemotherapeutic agents in initial studies but showed significant cardiotoxicity at therapeutic dosages. Combining Pim2 inhibition with GSK3 inhibition in TRAF3 low or negative BCLs could allow use of a lower dose with fewer off-target effects. Such studies of combination treatments will be an important priority going forward.

The enhanced loss of Mcl1 in 9-ING-41-treated BCL cell lines with low or no TRAF3 is of particular interest. GSK3 activity can increase or decrease cellular Mcl1 levels. GSK3 promotes Mcl1 translation via pathways that involve the kinases Akt and Erk, but it can also directly phosphorylate Mcl1 at key residues that target it for ubiquitination and degradation [41]. Translation-level loss of Mcl1 and cell death upon GSK3 inhibition was recently reported for Acute Myeloid Leukemia (AML) cell lines [42]. This may also apply to BCL cells, although it should be noted that TRAF3-mediated regulation of Mcl1 occurs at the transcriptional level. Nuclear TRAF3 recruits TRAF2 to the nucleus and facilitates poly-ubiquitination of the transcription factor CREB, leading to its degradation and thus interfering with transcription of its target genes, including Mcl1 [24]. TRAF3-deficient B cells have increased Mcl1 resulting from increased nuclear CREB in the absence of TRAF3-facilitated degradation. A GSK3-inhibition-induced breakdown in Mcl1 production at the translational level would supersede enhanced Mcl1 transcription due to decreased TRAF3. BCL cell lines with low or no TRAF3 may be more susceptible to apoptosis induced by GSK3 inhibition if they have developed a particular reliance on Mcl1 for survival. In this regard, it is relevant that several TRAF3-regulated signaling pathways in B cells lead to inhibitor resistance in primary B cells while conferring increased sensitivity to inhibitors in malignant B cells; these include Pim2 and Btk and suggest ‘oncogene addiction’ to such pathways in TRAF3-deficient malignant B cells.

## 5. Conclusions

We here show a relationship between GSK3 activation status and TRAF3 protein level in BCL-derived cell lines that has the potential to allow more accurate prediction of responders to GSK3 inhibitor therapy. TRAF3 low or negative BCL cell lines showed much higher susceptibility to GSK3 inhibition, but GSK3 inhibition showed no effect on viability of non-transformed, resting B cells. These findings provide an important foundation for further studies of how TRAF3-regulated pathways exploited by malignant B cells for survival can be targeted indirectly for therapeutic benefit.

## Data Availability

All original data are within the manuscript.

## Supplementary Materials

The following supporting information can be downloaded at: https://www.mdpi.com/article/10.3390/cancers14205029/s1, Figure S1. A. Representative blot of TRAF3, GSK3β, and pGSK3β^S9^ in a panel of BCL cell lines with varying susceptibility to 9-ING-41 treatment, as demonstrated in Figure 1, or by Wu et al. (ref. [19] in main text). AB199-1 and AB12-12 were GSK3-deficient subclones of OCI-Ly1, described by Wu et al. [19]. B. Graph of amount of TRAF3 vs ratio of inactive (pGSK3β^S9^): total pGSK3β from data in A. C. Uncropped blots from panel A. D. Quantification of blots in panel A. Figure S2: Susceptibility of BCL cell lines with varying levels of TRAF3 to GSK3 inhibitor CHIR-99021. Figure S3: Uncropped blots from Figure 1. Figure S4: Densitometry of blots from Figure 1. Figure S5: Uncropped blots and densitometry from Figure 3. Figure S6: Uncropped blots from Figure 4. Figure S7: Densitometry of blots from Figure 4.

## References

[1] Shaffer A.L., Young R.M., Staudt L.M. (2012). Pathogenesis of Human B Cell Lymphomas. Annu. Rev. Immunol..

[2] Shi J.H., Sun S.C. (2018). Tumor Necrosis Factor Receptor-Associated Factor Regulation of Nuclear Factor κB and Mitogen-Activated Protein Kinase Pathways. Front. Immunol..

[3] Bishop G.A., Stunz L.L., Hostager B.S. (2018). TRAF3 as a Multifaceted Regulator of B Lymphocyte Survival and Activation. Front. Immunol..

[4] Xie P., Stunz L.L., Larison K.D., Yang B., Bishop G.A. (2007). Tumor necrosis factor receptor-associated factor 3 is a critical regulator of B cell homeostasis in secondary lymphoid organs. Immunity.

[5] Lin W.W., Yi Z., Stunz L.L., Maine C.J., Sherman L.A., Bishop G.A. (2015). The adaptor protein TRAF3 inhibits interleukin-6 receptor signaling in B cells to limit plasma cell development. Sci. Signal..

[6] Mambetsariev N., Lin W.W., Wallis A.M., Stunz L.L., Bishop G.A. (2016). TRAF3 deficiency promotes metabolic reprogramming in B cells. Sci. Rep..

[7] Xie P., Poovassery J., Stunz L.L., Smith S.M., Schultz M.L., Carlin L.E., Bishop G.A. (2011). Enhanced Toll-like receptor (TLR) responses of TNFR-associated factor 3 (TRAF3)-deficient B lymphocytes. J. Leukoc. Biol..

[8] Chen Z., Krinsky A., Woolaver R.A., Wang X., Chen S.M.Y., Popolizio V., Xie P., Wang J.H. (2020). TRAF3 Acts as a Checkpoint of B Cell Receptor Signaling to Control Antibody Class Switch Recombination and Anergy. J. Immunol..

[9] Moore C.R., Liu Y., Shao C., Covey L.R., Morse H.C., Xie P. (2012). Specific deletion of TRAF3 in B lymphocytes leads to B-lymphoma development in mice. Leukemia.

[10] Bushell K.R., Kim Y., Chan F.C., Ben-Neriah S., Jenks A., Alcaide M., Fornika D., Grande B.M., Arthur S., Gascoyne R.D. (2015). Genetic inactivation of TRAF3 in canine and human B-cell lymphoma. Blood.

[11] Braggio E., Keats J.J., Leleu X., Van Wier S., Jimenez-Zepeda V.H., Valdez R., Schop R.F.J., Price-Troska T., Henderson K., Sacco A. (2009). Identification of Copy Number Abnormalities and Inactivating Mutations in Two Negative Regulators of Nuclear Factor-κB Signaling Pathways in Waldenström’s Macroglobulinemia. Cancer Res..

[12] Keats J.J., Fonseca R., Chesi M., Schop R., Baker A., Chng W.-J., Van Wier S., Tiedemann R., Shi C.-X., Sebag M. (2007). Promiscuous Mutations Activate the Noncanonical NF-κB Pathway in Multiple Myeloma. Cancer Cell.

[13] Annunziata C.M., Davis R.E., Demchenko Y., Bellamy W., Gabrea A., Zhan F., Lenz G., Hanamura I., Wright G., Xiao W. (2007). Frequent Engagement of the Classical and Alternative NF-κB Pathways by Diverse Genetic Abnormalities in Multiple Myeloma. Cancer Cell.

[14] Xie P., Hostager B.S., Bishop G.A. (2004). Requirement for TRAF3 in signaling by LMP1 but not CD40 in B lymphocytes. J. Exp. Med..

[15] Bangalore-Prakash P., Stunz L.L., Mambetsariev N., Whillock A.L., Hostager B.S., Bishop G.A. (2017). The oncogenic membrane protein LMP1 sequesters TRAF3 in B-cell lymphoma cells to produce functional TRAF3 deficiency. Blood Adv..

[16] Manning B.D., Toker A. (2017). AKT/PKB Signaling: Navigating the Network. Cell.

[17] Hoffmeister L., Diekmann M., Brand K., Huber R. (2020). GSK3: A Kinase Balancing Promotion and Resolution of Inflammation. Cells.

[18] Augello G., Emma M.R., Cusimano A., Azzolina A., Montalto G., McCubrey J.A., Cervello M. (2020). The Role of GSK-3 in Cancer Immunotherapy: GSK-3 Inhibitors as a New Frontier in Cancer Treatment. Cells.

[19] Wu X., Stenson M., Abeykoon J., Nowakowski K., Zhang L., Lawson J., Wellik L., Li Y., Krull J., Wenzl K. (2019). Targeting glycogen synthase kinase 3 for therapeutic benefit in lymphoma. Blood.

[20] Harrington C.T., Sotillo E., Robert A., Hayer K.E., Bogusz A.M., Psathas J., Yu D., Taylor D., Dang C.V., Klein P. (2019). Transient stabilization, rather than inhibition, of MYC amplifies extrinsic apoptosis and therapeutic responses in refractory B-cell lymphoma. Leukemia.

[21] Jellusova J., Cato M.H., Apgar J.R., Ramezani-Rad P., Leung C.R., Chen C., Richardson A.D., Conner E.M., Benschop R.J., Woodgett J.R. (2017). Gsk3 is a metabolic checkpoint regulator in B cells. Nat. Immunol..

[22] Lee J., Park H., Lim J., Jin H.-S., Park Y., Jung Y.-J., Ko H.-J., Yoon S.-I., Lee G.-S., Kim P.-H. (2021). GSK3 Restrains Germinal Center B Cells to Form Plasma Cells. J. Immunol..

[23] Thornton T.M., Delgado P., Chen L., Salas B., Krementsov D., Fernandez M., Vernia S., Davis R.J., Heimann R., Teuscher C. (2016). Inactivation of nuclear GSK3β by Ser389 phosphorylation promotes lymphocyte fitness during DNA double-strand break response. Nat. Commun..

[24] Mambetsariev N., Lin W.W., Stunz L.L., Hanson B.M., Hildebrand J.M., Bishop G.A. (2016). Nuclear TRAF3 is a negative regulator of CREB in B cells. Proc. Natl. Acad. Sci. USA.

[25] Whillock A.L., Mambetsariev N., Lin W.W., Stunz L.L., Bishop G.A. (2019). TRAF3 regulates the oncogenic proteins Pim2 and c-Myc to restrain survival in normal and malignant B cells. Sci. Rep..

[26] Kim K.J., Kanellopoulos-Langevin C., Merwin R.M., Sachs D.H., Asofsky R. (1979). Establishment and characterization of BALB/c lymphoma lines with B cell properties. J. Immunol..

[27] Menezes J., Leibold W., Klein G., Clements G. (1975). Establishment and characterization of an Epstein-Barr virus (EBC)-negative lymphoblastoid B cell line (BJA-B) from an exceptional, EBV-genome-negative African Burkitt’s lymphoma. Biomedicine.

[28] Tompkins V.S., Han S.S., Olivier A., Syrbu S., Bair T., Button A., Jacobus L., Wang Z., Lifton S., Raychaudhuri P. (2013). Identification of candidate B-lymphoma genes by cross-species gene expression profiling. PLoS ONE.

[29] Dyer M.J., Fischer P., Nacheva E., Labastide W., Karpas A. (1990). A new human B-cell non-Hodgkin’s lymphoma cell line (Karpas 422) exhibiting both t (14;18) and t(4;11) chromosomal translocations. Blood.

[30] Tweeddale M.E., Lim B., Jamal N., Robinson J., Zalcberg J., Lockwood G., Minden M.D., Messner H.A. (1987). The presence of clonogenic cells in high-grade malignant lymphoma: A prognostic factor. Blood.

[31] Klein E., Klein G., Nadkarni J.S., Nadkarni J.J., Wigzell H., Clifford P. (1968). Surface IgM-kappa specificity on a Burkitt lymphoma cell in vivo and in derived culture lines. Cancer Res..

[32] Klein G., Lindahl T., Jondal M., Leibold W., Menézes J., Nilsson K., Sundström C. (1974). Continuous lymphoid cell lines with characteristics of B cells (bone-marrow-derived), lacking the Epstein-Barr virus genome and derived from three human lymphomas. Proc. Natl. Acad. Sci. USA.

[33] Saiki O., Ralph P. (1983). Clonal differences in response to T cell replacing factor (TRF) for IgM secretion and TRF receptors in a human B lymphoblast cell line. Eur. J. Immunol..

[34] Krangel M.S., Orr H.T., Strominger J.L. (1979). Assembly and maturation of HLA-A and HLA-B antigens in vivo. Cell.

[35] Wallis A.M., Wallace E.C., Hostager B.S., Yi Z., Houtman J.C.D., Bishop G.A. (2017). TRAF3 enhances TCR signaling by regulating the inhibitors Csk and PTPN22. Sci. Rep..

[36] Ko R., Park J.H., Ha H., Choi Y., Lee S.Y. (2015). Glycogen synthase kinase 3β ubiquitination by TRAF6 regulates TLR3-mediated pro-inflammatory cytokine production. Nat. Commun..

[37] Edwards S.K., Han Y., Liu Y., Kreider B.Z., Liu Y., Grewal S., Desai A., Baron J., Moore C.R., Luo C. (2016). Signaling mechanisms of bortezomib in TRAF3-deficient mouse B lymphoma and human multiple myeloma cells. Leuk. Res..

[38] Zhu S., Jin J., Gokhale S., Lu A.M., Shan H., Feng J., Xie P. (2018). Genetic Alterations of TRAF Proteins in Human Cancers. Front. Immunol..

[39] Karmali R., Chukkapalli V., Gordon L.I., Borgia J.A., Ugolkov A., Mazar A.P., Giles F.J. (2017). GSK-3β inhibitor, 9-ING-41, reduces cell viability and halts proliferation of B-cell lymphoma cell lines as a single agent and in combination with novel agents. Oncotarget.

[40] Whillock A.L., Ybarra T.K., Bishop G.A. (2021). TNF receptor-associated factor 3 restrains B-cell receptor signaling in normal and malignant B cells. J. Biol. Chem..

[41] Senichkin V.V., Streletskaia A.Y., Gorbunova A.S., Zhivotovsky B., Kopeina G.S. (2020). Saga of Mcl-1: Regulation from transcription to degradation. Cell Death Differ..

[42] Lee Y.C., Shi Y.J., Wang L.J., Chiou J.T., Huang C.H., Chang L.S. (2021). GSK3β suppression inhibits MCL1 protein synthesis in human acute myeloid leukemia cells. J. Cell. Physiol..

