# Supplementary material for: The Tumor Suppressor Protein TRAF3 Modulates GSK3 Activity and Susceptibility of B Lymphoma Cells to GSK3 Inhibition
Source: Cancers (Basel). 2022 Oct 14;14(20):5029. doi: 10.3390/cancers14205029 (PMC9599470; doi:10.3390/cancers14205029)
Supplement: Supplementary file 1 [file cancers-14-05029-s001.zip › cancers-1910855-supplementary.pdf]

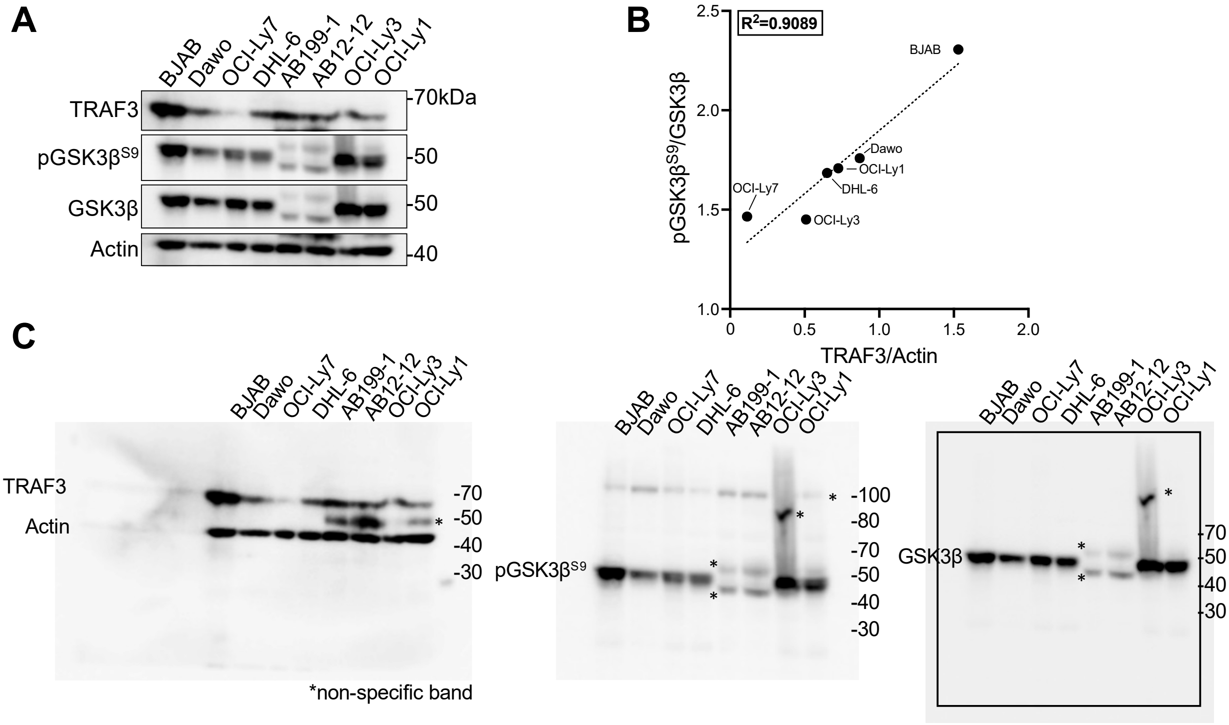

D

	TRAF3	Actin	pGSK3b	GSK3b	pGSK3b/ GSK3b	TRAF3/ Actin
**BJAB**	870058	568724	1425957	618390	2.30592	1.52984
**Dawo**	366235	421221	700245	398095	1.75899	0.86946
**OCI-Ly7**	51096.5	450484	728780	497448	1.46504	0.11343
**DHL6**	336728	517826	677805	402417	1.68433	0.65027
**AB199-1**	not included in analysis					
**AB12-12**						
**OCI-Ly1**	353095	486630	1238476	725064	1.70809	0.72559
**OCI-Ly3**	297263	583655	867950	598581	1.45001	0.50931

Figure S1. A. Representative blot of TRAF3, GSK3β, and pGSK3β^{S9} in a panel of BCL cell lines with varying susceptibility to 9-ING-41 treatment, as demonstrated in Figure 1, or by Wu et al. (ref 19 in main text). AB199-1 and AB12-12 were GSK3-deficient subclones of OCI-Ly1, described by Wu et al. [19]. B. Graph of amount of TRAF3 vs ratio of inactive (pGSK3β^{S9}): total pGSK3β from data in A. C. Uncropped blots from panel A. D. Quantification of blots in panel A.

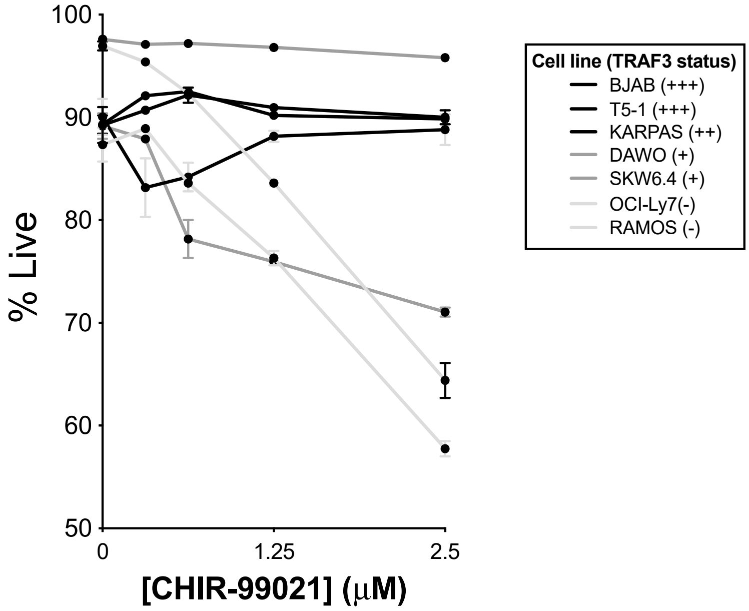

Figure S2. Survival of BCL cell lines after 72hr incubation with indicated concentrations of CHIR-99021. Data are from two independent replicates, graphed as mean ± SEM.

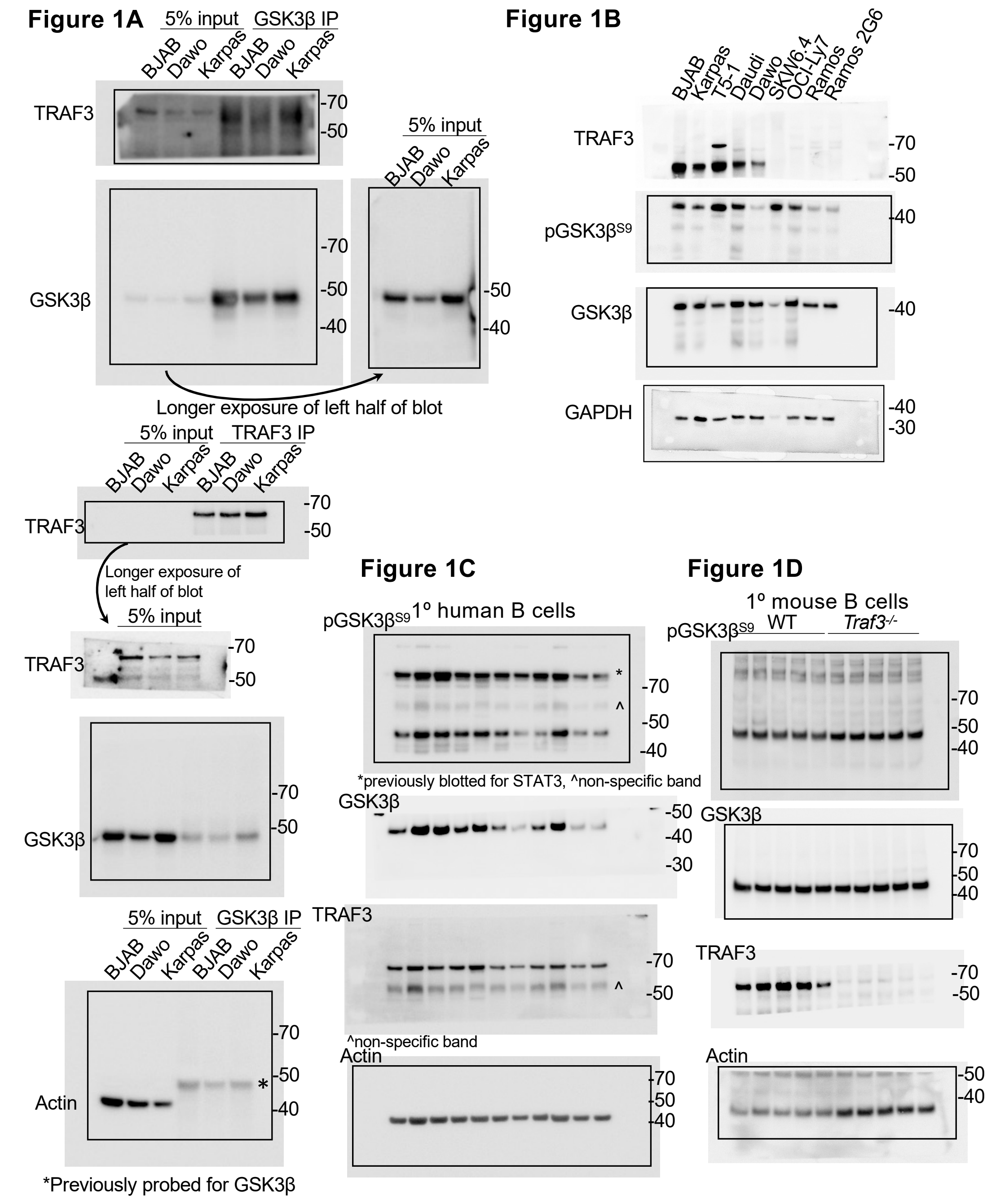

Figure S3. Uncropped blots from Figure 1.

Figure 1A

Sample		GSK3b	TRAF3 Full blot	TRAF3 Half blot
Input	BJAB	314187.7	not detected	157391.9
	Dawo	251356.1		137958.1
	Karpas	347476.7		139773.5
TRAF3 IP	BJAB	168029.3	302273.1	covered
	Dawo	150118.8	273091.7	
	Karpas	181945.4	314300.8	

Sample		TRAF3	GSK3b Full blot	GSK3b Half blot	Actin
Input	BJAB	257391.9	122945	244066.6	426950.4
	Dawo	237958.1	118530	201282.5	286104.7
	Karpas	239773.5	136115.6	294367.5	208593
GSK3b IP	BJAB	302273.1	325840.4	covered	not detected
	Dawo	273091.7	261341.9		
	Karpas	314300.8	237803.6		

Figure 1B

	TRAF3	GAPDH	TRAF3/ GAPDH	pGSK3b	GSK3b	pGSK3b/ GSK3b
**BJAB**	1222539	529906.5	2.30708	988688.3	889206.1	1.11188
**Karpas**	490806.3	335376.8	1.46345	480393.9	724890	0.66271
**T5-1**	751061.9	445370.9	1.68637	1061594	739518.6	1.43552
**Daudi**	403613.6	422969.4	0.95424	796383.5	1017139	0.78296
**Dawo**	178753.8	473748.6	0.37732	223060	908757	0.24546
**SKW6.4**	15382.94	275388.4	0.05586	65075.66	273405.4	0.23802
**OCI-Ly7**	2769.92	447006.8	0.0062	592310.1	982422.8	0.60291
**Ramos**	217.52	476580.2	0.00046	253035.3	873692.7	0.28962
**Ramos2G6**	1910.16	481778.9	0.00396	252959.4	905167.2	0.27946

Figure 1D & F

		pGSK3b	GSK3b	TRAF3	Actin	pGSK3b/ GSK3b	TRAF3/ Actin
WT 1º mouse B cells	1	363038.9	480891.5	111009.5	590005.4	0.754929	0.18815
	2	398226.3	328875.4	147892.8	409334.4	1.210873	0.361301
	3	341117.4	324900.9	178374.8	489281	1.049912	0.364565
	4	404738.1	391016.5	137184.8	510864.1	1.035092	0.268535
	5	407240.3	391619.2	73103.99	520856	1.039888	0.140354
Traf3-/- 1º mouse B cells	1	430590.5	428295.1	2079.06	604416.5	1.005359	0.00344
	2	435278.8	415320.6	7601.88	673447.2	1.048055	0.011288
	3	421358.7	422711.4	6300.9	665870.4	0.9968	0.009463
	4	450080.9	402043	6117.95	655343.1	1.119484	0.009335
	5	487121.2	437637.9	7840.08	641356.4	1.113069	0.012224
1º human B cells	1	619573.3	244611.9	168823.4	200765.3	2.532883	0.840899
	2	1010051	369478.4	191692.4	234235.1	2.733723	0.818376
	3	861953	319236.5	168839.4	218178.4	2.700045	0.773859
	4	639143.5	268821.6	169838.5	202184.8	2.377575	0.840016
	5	731160.4	299761.9	184622.6	199460.9	2.439137	0.925608
	6	520542.7	239449.2	149187.7	188561.6	2.173917	0.791188
	7	405526.7	166092.8	137518.5	173207.6	2.441567	0.793952
	8	544024.4	181305	152700.9	189611.9	3.000603	0.805334
	9	751478.6	334507.4	163872.3	202910.5	2.246523	0.807609
	10	437787.3	178907.4	142014	180887.7	2.447006	0.785095
	11	430426.8	186509	150797	186324.6	2.307807	0.809324

Figure S4. Quantifcation of blots from Figure 1.

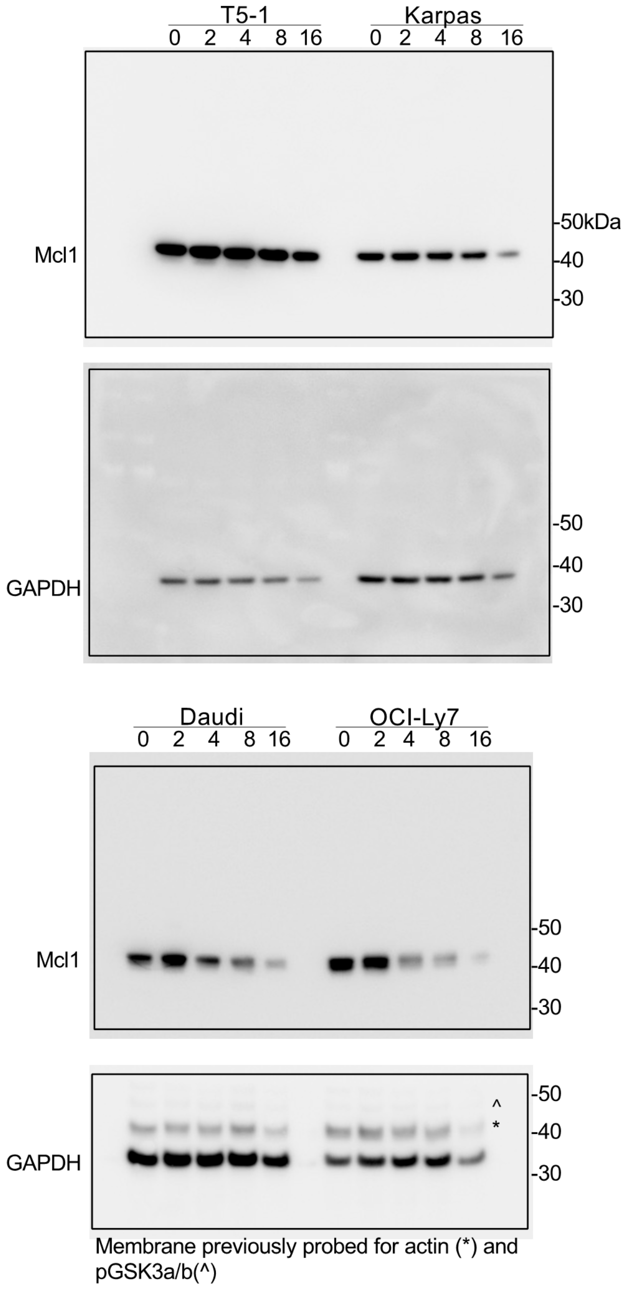

		Mcl1	GAPDH	Mcl1/ GAPDH
T5-1	0	1420795	333612.7	4.258815
	2	1703654	318325.9	5.351916
	4	1499489	247749.2	6.052446
	8	1397887	251777.9	5.552066
	16	905744	203767.6	4.444986
Karpas	0	697769	802787.9	0.869182
	2	719837	834120.6	0.862989
	4	644386	871346.6	0.739529
	8	556219	794079.5	0.700458
	16	324831	682122.1	0.476206
Daudi	0	476073	924115.8	0.515166
	2	622072	938034.6	0.663165
	4	407457	930105.1	0.438076
	8	364941	990912.8	0.368288
	16	256472	767329	0.33424
OCI-Ly7	0	637570	512094.1	1.245026
	2	643448	610063.2	1.054723
	4	328440	676999.6	0.48514
	8	276610	661361.7	0.418244
	16	212953	427635.7	0.497978

Figure S5. Uncropped blots and quantification from Figure 3A.

Figure 4A

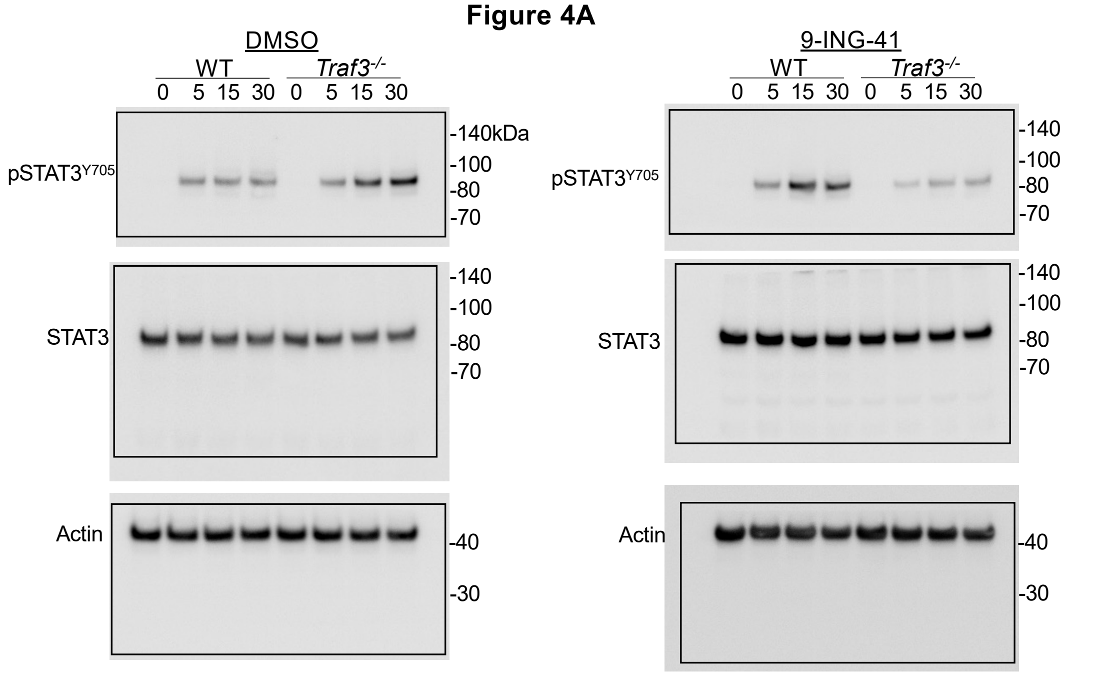

Figure 4C

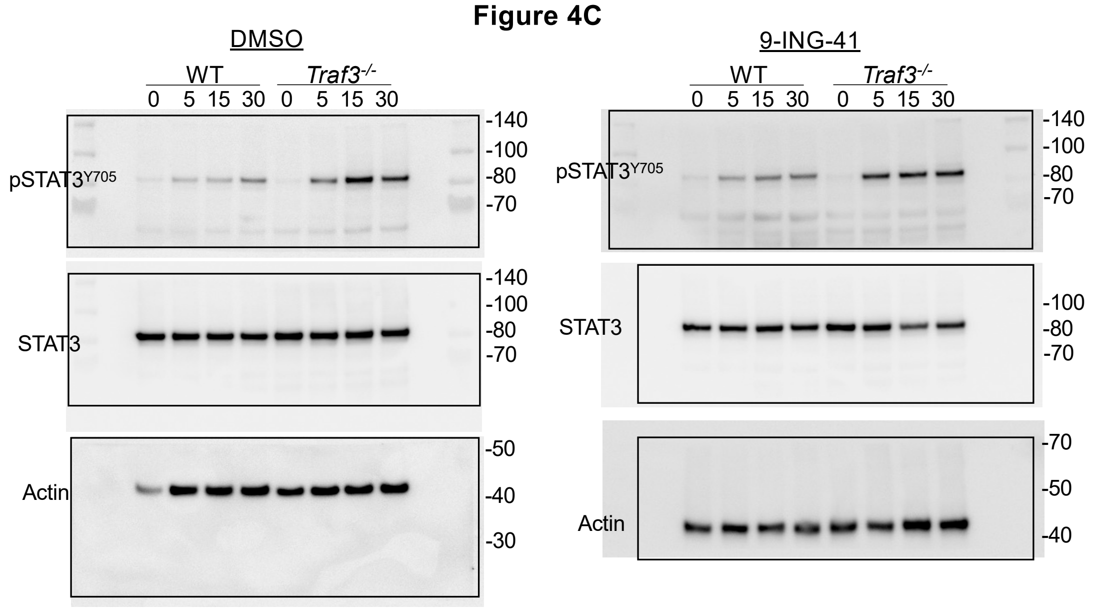

Figure S6. Uncropped blots from Figure 4.

		IL-6 (min)	pSTAT3	STAT3	Actin	pSTAT3 /STAT3	pSTAT3	STAT3	Actin	pSTAT3 /STAT3
**DMSO**	WT	0	13340.7	393023.8	680754.7	0.0339	43118.9	611872.1	161319.7	0.0705
		5	242903.5	406389.2	670514.3	0.5977	122994.3	636060.0	357441.6	0.1934
		15	264762.9	429064.5	918663.9	0.6171	118912.4	575418.1	398440.0	0.2067
		30	267999.8	402205.2	830371.2	0.6663	241274.5	633086.2	388776.5	0.3811
	Traf3-/-	0	19392.1	405983.5	821678.2	0.0478	23291.8	855907.6	378177.3	0.0272
		5	275014.8	406928.8	656397.3	0.6758	364865.1	857218.3	383248.4	0.4256
		15	415929.9	414933.1	671473.0	1.0024	586237.9	826422.9	404639.2	0.7094
		30	454533.5	398556.6	730549.7	1.1404	472917.8	861182.9	378397.5	0.5491
**9-ING-41**	WT	0	15603.7	610009.7	770926.0	0.0256	17665.3	653335.6	385927.4	0.0270
		5	206323.8	592985.2	870304.1	0.3479	190214.0	640774.9	393984.3	0.2968
		15	289150.3	614173.5	907207.1	0.4708	269195.4	681789.2	412058.0	0.3948
		30	351952.2	620811.5	982482.6	0.5669	281545.4	618890.7	418593.9	0.4549
	Traf3-/-	0	14994.0	596141.2	767278.2	0.0252	32292.8	803999.3	354041.9	0.0402
		5	222773.9	590011.5	748307.2	0.3776	627460.5	753981.8	389299.5	0.8322
		15	248531.9	595991.0	779846.1	0.4170	441557.7	473297.9	406808.7	0.9329
		30	255315.8	609743.9	722394.7	0.4187	431240.3	483579.7	401456.1	0.8918

Figure S7. Quantification of blots in Figure 4.
